# Lithium Isotope Separation Using the 15-Crown-5 Ether System and Laboratory-Made Membranes

**DOI:** 10.3390/ma18092016

**Published:** 2025-04-29

**Authors:** Andreea Maria Iordache, Ana Maria Nasture, Ramona Zgavarogea, Radu Andrei, Roxana Mandoc, Erdin Feizula, Rui Santos, Constantin Nechita

**Affiliations:** 1 ICSI Analytics Department, National Research and Development Institute for Cryogenics and Isotopic Technologies—ICSI, 4 Uzinei Street, 240050 Râmnicu Vâlcea, Romania; andreea.iordache@icsi.ro (A.M.I.); ramona.zgavarogea@icsi.ro (R.Z.); radu.andrei@icsi.ro (R.A.); roxana.mandoc@icsi.ro (R.M.); 2 ICSI Energy Department, National Research and Development Institute for Cryogenics and Isotopic Technologies—ICSI, 4 Uzinei Street, 240050 Râmnicu Vâlcea, Romania; ana.nasture@icsi.ro; 3 Biotechnologies Department, Faculty of Chemical Engineering and Biotechnologies, 313 Splaiul Independentei, 060042 Bucharest, Romania; erdin.feizula@analytik-jena.com; 4 Analytik Jena GmbH at CIQUP, Faculdade de Ciências da Universidade do Porto, Rua Campo Alegre 687, 4169007 Porto, Portugal; rui.santos@analytik-jena.com; 5 Department of Biometry, National Research Institute in Forestry Marin Dracea—INCDS, 128 Eroilor, 077190 Voluntari, Romania

**Keywords:** lithium isotope separation, electromigration process, ^6^Li enrichment

## Abstract

The enrichment of ^6^Li isotopes from a natural stage of 7.6% to above 59% is required for the development of next-generation green technologies capable of sustaining climate change mitigation and energy-mix targets. In this study, we developed two categories of custom laboratory-made organic membranes, membranes that were non-impregnated before electromigration (AI-1) and membranes impregnated with LiNT*f*_2_ (AI-2), to evaluate their performance in lithium isotope separation. Both types of membranes were exposed in synthesis to ionic liquid and crown ether. The objective of the study was to test the performance of membranes in separating lithium isotopes from a lithium-loaded organic phase in an aqueous solution with variable potentials and time intervals. The results show that the impregnated AI-2 membranes increased the enrichment of ^6^Li in the early stages, and the effect decreased after 25 h. The efficiency of lithium isotope enrichment was positively related to the potential profile applied, migration time, and concentration of organic solution in the anode chamber. The 0.5 mol/L Bis-(trifluoromethane) sulfonimide lithium salt (Li[NT*f*_2_]) with 0.1 M tetra butyl ammonium perchlorate (TBAP) in acetonitrile (CH_3_CN) ionic solution significantly improved Li isotope separation compared with an aqueous environment with higher salt concentrations. The maximum isotopic separation coefficient (α) for AI-1.2 (15-crown-5 ether and 1 mol/L LiNT*f*_2_ in TBAP solution after 48 h of electromigration) gradually increased to 1.0317. Our results demonstrated that in the laboratory-made setup described, the migration efficiency and Li isotope separation in the catholyte environment needed a minimum of 9 V and a migration time of 6 h, respectively; these values varied with the concentration of the organic solution in the anode chamber. The ability of laboratory-engineered membranes to impart isotope selectivity and enhance permselectivity or selectivity towards singly charged ions was demonstrated through the functionality of single-collector inductively coupled plasma mass spectrometry (ICP-MS). This technology is particularly valuable and commercially feasible for future lithium isotope research in nuclear technology.

## 1. Introduction

Lithium is a critical element with high electrochemical activity, low density, and high capacity for energy storage and release, and is widely used across various fields, such as energy technology, transportation, nuclear energy, metallurgy, chemistry, pharmaceuticals, and aerospace [1,2,3]. Lithium isotope properties enhance their value in future green technologies for clean nuclear energy production. With a natural abundance of only 7.5%, ^6^Li is a rare isotope that can absorb neutrons and produce tritium in nuclear reactions, causing it to be of interest in research, technology, and future technologies [4]. On the other hand, ^7^Li, which has a natural abundance of 92.5%, has valuable importance for electric vehicle propulsion, electrical equipment, and heavy water reactors, and its properties help reduce neutron absorption in water [5,6,7,8,9]. The separation of lithium isotopes, particularly ^6^Li and ^7^Li, remains a complex challenge in modern industry, with significant implications for the development of advanced technologies. Various separation techniques have been developed, each offering distinct advantages and limitations [3,10,11,12,13]. Although ^6^Li and ^7^Li share similar chemical properties, their unique nuclear characteristics underscore the importance of their separation and enrichment [14,15,16]. A notable approach was developed by Hoshino and Terai [17], who introduced a technique for separating lithium isotopes using a highly porous Teflon foil, 1 to 3 mm thick, as an organic membrane [17].

Electromigration is a relatively new technique for separating Li isotopes using ionic liquid systems. Using this approach, Hoshino et al. [10] achieved Li isotope separation via electrodialysis with an ionic-liquid-impregnated organic membrane in an aqueous solution, obtaining a notable separation coefficient of 1.4. This efficiency was attributed to the different mobilities of ^6^Li and ^7^Li ions within the ionic liquid medium. The advantages of ionic liquid–crown ether systems are being intensively studied as promising approaches for Li isotope separation through extraction processes [10,13,17,18]. For instance, Zhang et al. [19] used three different ionic liquids in electromigration, achieving striking results in isotope separation. They found notable diffusivity differences between ^6^Li and ^7^Li across various ionic liquids, thus enhancing separation efficiency. Sun et al. [11] reported a maximum single-stage separation coefficient of 1.046 by substituting conventional molecular solvents with ionic liquids in liquid–liquid extraction. Zhou et al. [20] developed a sustainable method for lithium isotope separation by doping mesoporous silicon with ionic liquids and benzo-15-crown-5. However, they observed that crown ether tended to leach from the doped silica material after immersion in ethanol for 36 h. In addition, 15-Crown-5 is a macrocyclic ether known to selectively bind lithium ions (Li⁺) due to its cavity size of about 1.7–2.2 Å. This precise fit makes the formation of stable complexes possible, and these have been utilized in Li isotope separation processes. In a separation system, 15-Crown-5 can preferentially interact with one Li isotope through selective binding, thus facilitating separation. Various studies reported that a separation system using 15-Crown-5 can transport Li^+^ between aqueous and organic phases, enabling effective isotope fractionation [21]. 15-crown-5-ether into dibutyl phthalate (DBP) in a Li^+^ detection membrane depends on several factors, including ion selectivity, membrane stability, and sensor performance [22].

The membrane-based process for Li separation is relatively novel and is easy to operate on a small scale, with low energy consumption, promising efficiency, and a low environmental footprint. The membranes on the market are widely available for Li harvesting technologies, such as recycling, which uses nanofilms, membrane electrodialysis, membrane absorption, and membrane solvent extraction. However, commercially available organic membranes used for separation are less accessible. They are subjected to various disadvantages, including availability and materials beyond the cost of purchase, which are affected by market fluctuations. One significant advantage of laboratory-made membranes is their customizability. Customization includes the integration of crown ethers for different periods in various combinations, which play a crucial role in selectively capturing lithium ions and can be produced on a small scale, allowing for rapid prototyping and testing of new designs without the need for large-scale manufacturing facilities. This also offers a cost-effective solution, which is especially valuable for niche applications such as Li isotope separation at an industrial scale [18,23,24,25]. Membranes impregnated with crown ethers help minimize cross-contamination between isotopes, ensuring higher purity levels of the desired isotope, which increases ^6^Li separation and enrichment efficiency.

In this study, we develop two groups of laboratory-made membranes, non-impregnated membranes with LiNT*f*_2_ before electromigration (AI-1) and impregnated membranes (AI-2), to evaluate their performance in lithium isotope separation. The membranes were characterized, tested, and assessed in the first stage to understand the mechanism behind isotope separation. The study aimed to test individual and combined interactions between several factors—such as membranes, voltage, migration time, and organic solutions—involved in the electromigration process. In addition, the experiments indicate the minimal thresholds of the parameters for efficient lithium ion migration in an electrolyte solution. The following hypotheses were adopted: (i) the membranes would exhibit varying degrees of performance, with potential failure, and (ii) by adjusting and combining different parameters, the impact of external factors on membrane properties and separation efficiency can be examined.

## 2. Materials and Methods

### 2.1. Chemical Reagents

Laboratory-made membranes for Li isotope separation require specialized materials that ensure selective permeability and durability. They are composed of polymers known for their stability and resistance to harsh chemical environments. In this study, polyvinyl chloride (PVC, Sigma-Aldrich, Munich, Germany), dibutyl phthalate (DBP, 99%, Sigma-Aldrich), 15-crown-5 (Sigma-Aldrich, Munich, Germany), and tetrahydrofuran (THF, Supelco, St. Louis, MO, USA) were selected as primary raw materials. Lithium carbonate (Li_2_CO_3_, ≥98%, IAEA, LSVEC, Vienna, Austria), anisol (99.8%), and ammonium chloride (NH_4_Cl, 99.999%) were used (Sigma-Aldrich, Munich, Germany). Additionally, ionic liquids, including 1-butyl-3-methylimidazolium bis (trifluoromethylsulfonyl)imide (Sigma-Aldrich, Buchs, Switzerland) and bis(trifluoromethane)sulfonimide lithium salt (LiNT*f*_2_, 99%), were obtained from Sigma-Aldrich, Buchs, Switzerland. All chemicals used in the electromigration experiments were of the highest purity. Other reagents included tetrabutylammonium perchlorate (Fluka Analytical, London, UK), perchloric acid (70–72%, Sigma-Aldrich, Germany), and nitric acid (Suprapur 65%, Sigma-Aldrich, Buchs, Switzerland). Tetrabutylammonium perchlorate and acetonitrile enhanced the electrochemical processes and provided high conductivity without interfering with the electrochemical reactions.

### 2.2. Experimental Apparatus

The concentrations of Li^+^ in the resulting samples were determined using a quadrupole inductively coupled plasma mass spectrometer (Q-ICP-MS, PlasmaQuantMS Elite, Analytik Jena, Jena, Germany) equipped with an autosampler (AIM 3300, A.I. Scientific, Clontarf, Australia) and collision reaction interface (iCRI) operating in the H_2_ and He modes for lithium isotope analysis. This setup was used to evaluate Li isotope separation effects and to measure isotope ratios (^6^Li/^7^Li) in the sample solutions. A multi-channel potentiostat with impedance (1A; model: Origalys, Rillieux-la-Pape, France), which was controlled with OrigaMaster 5 software (Evreux, France), was used to supply the electric field. Electrochemical cells with two platinum wire electrodes (1 mm diameter) were used, designated as the counter electrode (CE–Pt wire) and the working electrode (EL–Pt wire), for the experimental studies conducted in the ISO-Li project. Solution viscosity measurements were carried out with a Haake Viscotester iQ rheometer equipped with a Thermo Scientific Peltier temperature control system (Dreieich, Germany).

### 2.3. Membrane Synthesis

**Figure 1:**
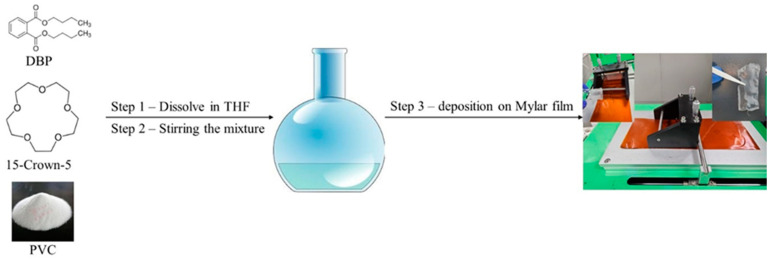
Graphical illustration of the Doctor Blade equipment for membrane production.

The Li^+^ detection membrane was synthesized by mixing 15-crown-5, dibutyl phthalate, and polyvinyl chloride (PVC) at a molar ratio of 1:0.523:0.31 in tetrahydrofuran (THF) as a dispersing solvent. The mixture was stirred for 8 h at 55 °C and kept below the boiling point of THF to achieve a homogenous solution. The membrane was then formed using Doctor Blade deposition equipment and allowed to rest for 24 h at room temperature for THF evaporation (Figure 1). Two membranes were prepared with different molar ratios of 15-crown-5 to DBP of 1:1 (AI-1) and 2:1 (AI-2). The resulting membranes had a thickness of 0.039 mm.

### 2.4. Laboratory-Made Membrane Characterization

This study developed and tested two groups of laboratory-made membranes, AI-1 and AI-2, which were exposed to ionic liquid and crown ether in synthesis (in the first manufacturing stage). The AI-2 membranes were subjected to double the amount of crown ether with a ratio of 2:1. The AI-1 was non-impregnated with LiNT*f*_2_ before electromigration (hereafter, non-impregnated), and AI-2 was impregnated with LiNT*f*_2_ before electromigration (hereafter, impregnated); they were further adjusted by varying the crown ether content to evaluate their efficacy in lithium isotope separation. A detailed description of the experimental setup for membrane production and electromigration is presented in the following.

The solution viscosity was measured using a Haake Viscotester iQ rheometer (Karlsruhe, Germany) with Thermo Scientific Peltier temperature control, employing cylindrical coaxial geometries with a 10 mL sample volume. Flow curves were recorded at 25 °C, with a logarithmic distribution over 120 s and an integration time of 5 s, covering a shear rate ranging from 10^−1^ s^−1^ to 1500 s^−1^. Data were analyzed using the power law model according to Equation (1). Broadband dielectric measurements were conducted with a Novocontrol Concept 40 high-resolution Alpha dielectric analyzer (Montabaur, Germany), covering a frequency range from 10^−1^ Hz to 10^7^ Hz and a temperature range of −150 °C to 200 °C; this was managed by a Quattro Cryosystem (Montabaur, Germany) with a temperature stability of up to 0.3 K in a dry nitrogen atmosphere. The polyelectrolyte thin film was placed between two polished gold electrodes (25 and 15 mm) to create a parallel-plate capacitor cell. These sandwich samples were then positioned in the Novocontrol spectrometer, where the dielectric permittivity and conductivity were measured isothermally at temperature intervals of 10 K during cooling and 5 K during heating, ensuring reproducibility. An AC voltage amplitude of 0.05 V was applied across the whole temperature range and frequency spectrum. Additionally, these polymers could be modified with functional groups or additives, such as crown ethers, to enhance Li^+^ affinity, particularly for the separation of ^6^Li and ^7^Li isotopes. The impregnation of laboratory-made membranes is essential in enriching the target isotope ^6^Li. Therefore, we tested two types of impregnation for all membranes during their creation and conducted hyper-impregnation on the second group of membranes before electromigration (Table 1). After manufacturing the membranes, we tested their performance in the electromigration process, particularly in terms of the properties described in the section on membrane optimization.

(1) $$\eta \left(ẏ\right)=Kẏfb-1$$

where η (Pa·s) is the viscosity as a function of the shear velocity, ẏ (s^−1^), K is the flow consistency index, and fb indicates the flow behavior index.

### 2.5. Electromigration and Analytical Experiments

**Figure 2:**
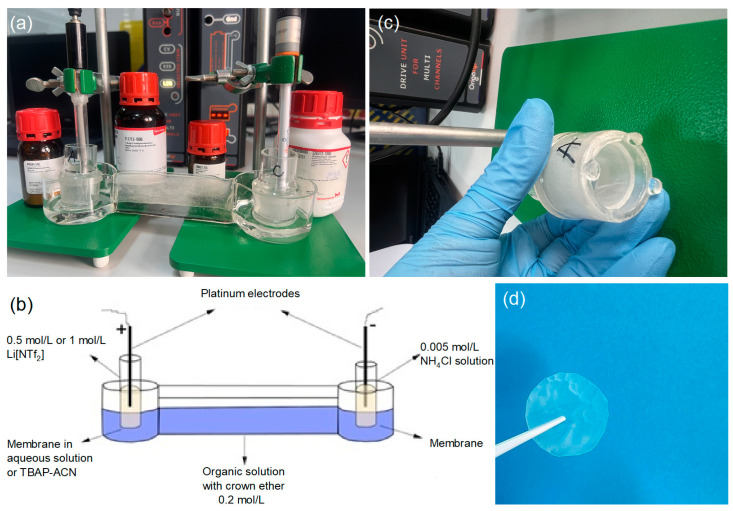
The experimental electromigration setup was manufactured in the laboratory using borosilicate glass to avoid contamination that would negatively influence Li separation. Panel (**a**) illustrates the electrochemical cells with two electrodes (CE–Pt wire and EL–Pt wire electrodes), membranes placed in the anode and cathode chambers, the electrochemical system in the OrigaLys 5 software, and the chemicals. Panel (**b**) shows the electromigration separation of lithium isotopes in the system with crown ether. Panel (**c**) shows the membrane fixed in the anode chamber. Panel (**d**) shows the membrane after the electromigration process.

The ionic liquid 1-butyl-3-methylimidazoliu bis((trifluoromethyl)sulfonyl)imide was mixed with anisole in a ratio of 7:3, and 0.2 mol/L of 15-crown-5 ether was added to create the organic solution, which was then placed in the intermediate tank. The anode solution was prepared using different concentrations (0.5 mol/L and 1 mol/L) of lithium bis(trifluoromethane)sulfonimide (LiNT*f*_2_) in aqueous and tetrabutylammonium perchlorate (TBAP) solution and placed in the anode tank. The cathode solution consisted of a 0.005 mol/L aqueous solution of NH_4_Cl and was placed in the cathode tank. Two laboratory-made membranes impregnated with 15-crown-5 and DBP in different molar ratios, 1:1 (AI-1) and 2:1 (AI-2), were used to separate the anode, organic, and cathode solutions. A platinum wire electrode with a 1 mm diameter was placed in both the anode and cathode solutions. An electric field was applied using a DC power supply, Origalys potentiostat (Rillieux-la-Pape, France), at different potentials (3–15 V). Samples from the anodic and cathodic solutions were collected at 2, 6, 20, 25, and 48 h intervals. In our experiment, electrochemical studies on the lithiation/delithiation process were conducted using the chronoamperometric method at a constant electric potential. Table 1 provides the operating conditions under which optimization tests were performed for the electrochemical lithiation/delithiation process. The chronoamperometric method was applied over 48 h. The experiment evaluated each membrane with different factor variations, such as lithium salt concentration, membrane type, working environment, and applied electric potential, to optimize the electrochemical procedure. Additionally, experiments were conducted in aqueous and organic (0.1 M TBAP/CH_3_CN) environments. The electromigration process was performed using a manufactured experimental setup (Figure 2).

### 2.6. Sample Analysis Using Isotopic Ratio Measurements

Organic matter was collected through a five-step back-extraction from the organic phase into a hydrolytic acid solution to evaluate the Li^+^ concentration. The samples were dry-extracted in a sand bath with concentrated nitric acid and perchloric acid in a 4:1 volume ratio at 195 °C with a heating power of 70 W. After dry digestion, the resulting solution was clear and transparent, allowing for the accurate testing of the Li^+^ concentration and Li isotope values. Sample digestion was conducted at the Isotope Metals Laboratory within the ICSI Analytics Department at the ICSI Institute. Samples of 1 mL were taken from both the anode and cathode solutions at intervals of 2, 6, 20, 25, and 48 h. For precise Li isotope measurement, we focused on purifying lithium by eluting other elements (e.g., Na and K) that could interfere with the Li isotope ratios. The chromatography system was designed in two stages to ensure high sample loading and a fixed Li elution range. In this experiment, BioRadTM Econo-Pac columns (1.5 cm ID × 12 cm H, polypropylene) were used following the starting procedure of a volume of 1 mL of 0.7 mol/L HNO_3_ that was eluted through the columns and cleaned with 50 mL of 6N HCl. In the recovered Li solutions, we analyzed the concentrations of ^23^Na, ^39^K, ^6^Li, and ^7^Li. Aliquots of each sample were prepared in 0.7 M HNO_3_ (*v*/*v*) containing 1, 5, 25, 50, and 100 ppb of Li for quantitative Q-ICP-MS analyses, along with 10 ppb of LSVEC reference material for Li isotope ratio measurements. Then, 45 mL of Li fractions were collected after column separation and quantitatively analyzed for ^6^Li, ^7^Li, ^23^Na, and ^39^K using Q-ICP-MS Plasma-Quant MS Elite (Analytik Jena, Jena, Germany). A standard extraction ratio (*E*) was employed to quantify the total ion content transferred into the organic phase, represented as the percentages of cation concentration in the aqueous phase before extraction (C_o_) and after extraction (C_e_), as detailed in Equation (2).

(2) $$E\left(\%\right)=\frac{C_{o}-C_{e\;}}{C_{o\;}}\times 100\%$$

We used a quadrupole inductively coupled plasma mass spectrometer (Q-ICP-MS, PlasmaQuant MS Elite, Analytik Jena, Jena, Germany) and the sample–standard bracketing method. Each sample was bracketed with the L-SVEC Lithium Carbonate standard, and the resulting measurements were compared with the standard according to the calculation outlined in Equation (3). The optimized instrumental setup was used to conduct high-precision lithium isotope measurements (2RSD = ±0.30‰) for natural carbonate samples. The organic solution in the (^6^Li/^7^Li) standard, which reflects the lithium isotope abundance ratio, for the L-SVEC standard is 0.08215 ± 0.00023 (IAEA-LSVEC Lithium Carbonate, Vienna, Austria). The Li isotope ratio (δ^7^Li) was calculated by standardizing the tested sample against the LSVEC standard, which was lithium carbonate (Li_2_CO_3_) manufactured by the National Institute of Standards and Technology [26].

The efficiency of the isotopic enrichment was quantified using the separation factor (α), which reflects the distribution of the desired isotope between two phases: the enriched and depleted phases. The key parameter for characterizing this separation process is the single-stage separation coefficient, denoted as α, or the alpha factor (Equation (4)). The separation factor indicates the degree of fractionation achieved in each phase after an enrichment step [27]. By definition, this equals the isotopic abundance ratio in each phase after an enrichment step.

(3) δ7Li·%, LSVEC=(L6i/L7i)sample(L6i/L7i)standard L−SVEC−1×1000

where (^6^*Li*/^7^*Li*)*_sample_* represents the Li isotope abundance ratio in samples extracted during the experiment, while (^6^*Li*/^7^*Li*)*_standard_* _L_-_SVEC_ denotes the Li isotope abundance ratio in the L-SVEC standard. The resulting *δ*^6^*Li* reflects the Li isotopic values from the experiment. The separation coefficient, α, was used to indicate the Li isotope separation effect during the electromigration process and was calculated using Equation (4).

(4) α= (Li6 / Li7)cathode  (Li6 / Li7)anode 

where [^6^Li]/[^7^Li] represents the Li isotope abundance ratio at the cathode phase and the anode phase in the two liquid separation processes.

## 3. Optimization Process

### 3.1. Voltage

A lower ligand concentration was used to reduce the process cost. The chronoamperometric curves revealed that increasing the concentration did not significantly influence the lithiation/delithiation process. Thus, the aqueous medium had a lower current variation (0–1 nA) than the organic environment, which exhibited a broader current range from −30 nA to +30 nA. The applied electric potential was also varied between 3 and 15 V. In this case, it was observed that the higher potential of 15 V was found to favor the lithiation/delithiation process, as demonstrated by the high current variation, ranging from −45 nA to +45 nA, compared with the smaller current range (0–1 nA) observed at 3 V.

### 3.2. Electrochemical Performance

**Figure 3:**
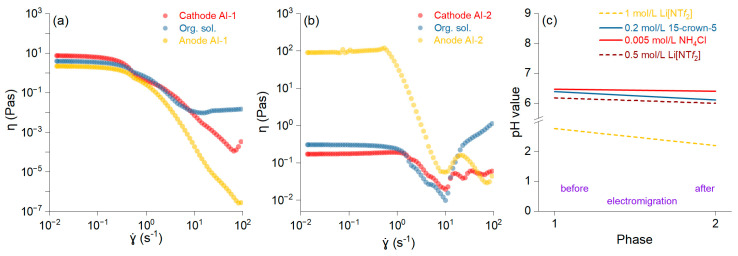
Shear viscosity versus shear rate for the anolyte, organic, and catholyte solutions and membranes AI-1 (**a**) and AI-2 (**b**). pH values for all solutions before and after the electromigration process (**c**).

The fluidity of the samples is depicted as the shear rate versus the shear viscosity, and it was higher in AI-2 (Figure 3b) than in AI-1 (Figure 3a). The connected network structures within the slurry break down at higher shear rates, leading to a thinning effect and a smoother viscosity profile. The increase in viscosity after a shear rate of 10^1^ is likely due to the decreasing pH of the solution (Figure 3c). Slurry viscosities decrease with increasing shear rates, exhibiting non-Newtonian flow properties [28]. The pH reduction results in the protonation of specific groups, which renders particles more hydrophobic, thereby contributing to the increased viscosity [29]. The changes in the viscosity affect the electromigration process and reduce the enrichment factor. It was noted that when using DBP with low ionic conductivity, the improved conductivity was mainly attributed to its interaction with crown ether molecules [30].

### 3.3. Relationship Between Ionic Conductivity and Temperature

**Figure 4:**
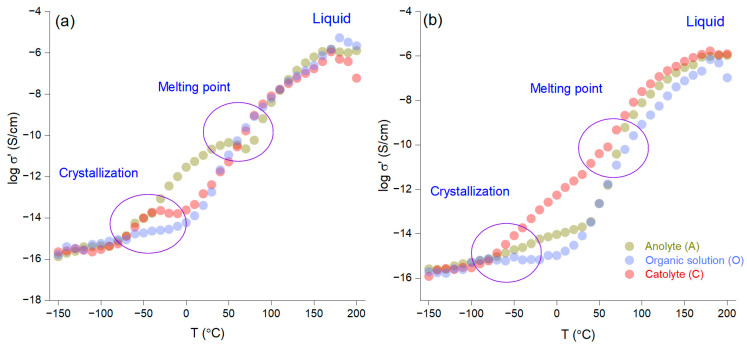
Ionic conductivity as a function of temperature for the AI-1 (**a**) and AI-2 (**b**) membranes.

Figure 4a,b present a kinetic analysis of the AI-1 and AI-2 membranes as a function of temperature, with an isochron representation that was carried out in 10 °C increments from −150 °C to 200 °C during the heating phase. The results show that quantitative crystallization occurs between −50 °C and 0 °C, while the melting point is observed between 50 °C and 100 °C. Notably, the non-impregnated membrane with LiNT*f*_2_ before electromigration (AI-1) demonstrates superior ionic conductivity to that of AI-2 across the entire temperature range. This difference is likely due to the higher concentration of crown ether in AI-1, which increases the number of crown ether molecules within the organic phase and subsequently enhances the number of phthalate molecules bound to the crown ether.

### 3.4. Membrane Permeability and Stability in Time

The permittivity of the fresh membrane gradually decreases with increasing frequency. This decline (higher in AI-2) occurs because, at high frequencies, the movement and rotation of dipoles are not aligned with changes in the electric field, with some dipoles even ceasing to reverse entirely. The permittivity decreases as the concentration of crown ether increases due to the presence of multiple binding sites within the ether’s frameworks, contributing to higher polarizability. The ion dynamics displayed in the Ɛ″ spectra suggest that dipolar-like relaxations are associated with the dielectric constant. Crown ether interactions with cations vary depending on thermodynamic and kinetic constraints influenced by the solvent’s nature and composition. In a secondary analysis, the dielectric spectra of the membrane across frequencies, and temperature intervals at the anode for AI-1 and AI-2 were measured to assess potential degradation following initial exposure to the electrolytic environment. The results confirm the superior dielectric properties of the AI-1 membrane. Lastly, the dielectric spectra were evaluated for AI-1 and AI-2 under identical frequency and temperature conditions at the cathode to detect possible signs of significant degradation. The membranes were nearly stable in this experiment, with unchanged properties. The literature shows that permittivity increases with rising temperatures as a result of the enhancement of high-temperature resistance in metallized film capacitors [31]. Conductance measurements of crown ethers, in turn, offer insights into ion–solvent and ion–ion interactions in the presence of crown ethers [32].

## 4. Results and Discussion

To conduct our experiment, an electromigration laboratory system was created to separate Li isotopes using solution–organic and solution–aqueous environments with the lithium salt solution in the anode chamber. The initial Li salt solution concentration was 7000 mg/L. After the experiment, it was measured at 6491 mg/L in the anode chamber, indicating that only 0.19% of the Li^+^ migrated from the anode solution to the organic phase in the case of the AI-2 membranes. Higher Li^+^ migration at the level of 48% was achieved in the case of non-impregnated membranes with LiNT*f*_2_ before electromigration (AI-1). Our results show that the transfer of Li^+^ from the aqueous anode solution to the organic phase was highly restricted, which was likely due to the bulk organic solution impeding migration, with only 10.20 mg/L being detected from the total concentration in the case of AI-2. Li^+^ presence near the cathode chamber reached 16.51% at 48 h (AI-2), resulting in a low concentration in the cathode solution. The low Li^+^ migration efficiency of the AI-2 membrane could be associated with the high viscosity induced by the pH, which renders the membrane susceptible to structural degradation at high shear rates. Similar studies show that Li^+^ in the cathode solution depends on that from the organic solution, and the increasing time of electromigration promotes the increase in Li^+^ in the cathode solution [33]. An experiment evaluating the separation system with B12C4, B15C5, and B18C6 transfer catalysis in a LiCl solution showed that separation effectiveness was mainly influenced by the interface between the anolyte and organic solution; the electric field had no significant effect [34]. Separation forces are dynamic, and diffusion increases with the concentration gradient, while chelation negatively correlates with increasing Li^+^ concentrations in organic solutions [35]. Another study using a three-stage system of “LiCl aqueous solution (anolyte)|B12C4-[EMIm][NTf_2_] organic solution|NH_4_Cl aqueous solution (catholyte)” found that voltage and migration time significantly affect isotope separation [36].

**Figure 5:**
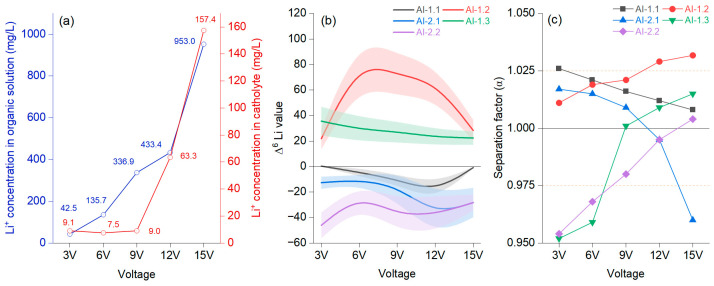
Average concentration of Li^+^ in the organic and cathode solutions (**a**); different voltages at a migration time of 25 h with 15-crown-5 and a LiCl concentration of 0.2 mol/L at the catholyte–organic solution interface, represented as a B-spline, with bands representing confidence intervals (**b**); separation factor (α) (**c**).

**Figure 6:**
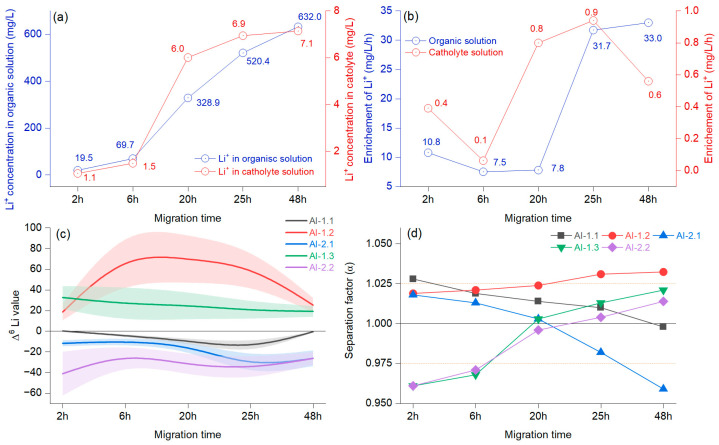
Average concentration of Li^+^ in the organic and cathode solutions at different migration times (**a**); average Li^+^ concentration changes over time (**b**); separation factor (α) represented as a B-spline, and bands represent confidence intervals (**c**); different migration times at 15 V with 15-crown-5 and a LiCl concentration of 0.2 mol/L (**d**).

Notably, results were achieved after modifying the system with a TBAP (tetra butyl ammonium perchlorate) solution in CH_3_CN and a Li salt solution in the anode chamber. From 7000 mg/L, the stock salt solution concentration was measured at only 6850 mg/L in the anode chamber, indicating a 0.20% migration rate. The Li^+^ in the organic solution near the cathode increased by 48.34% at 24 h, resulting in a higher concentration of Li^+^ in the cathode solution. The AI-1.1 membrane had no fractionation effect in the anode liquid–liquid environment, with no observed ^6^Li enrichment, which diminished until 25 h (Figure 5). For AI-1.2, the membrane showed the best performance with a positive enrichment factor at each evaluated interval, achieving the maximum at 25 h (Figure 6b). In the case of ^7^Li, the transfer occurred until 25 h, after which enrichment reached 52.37%, suggesting different migration rates for the two isotopes. Using a 0.5 mol/L Li[NT*f*_2_] (Bis-(trifluoromethane) sulfonimide lithium salt) solution with 0.1 M TBAP in CH_3_CN significantly improved lithium isotope separation compared with that in an aqueous environment with higher salt concentrations. Following digestion, the TBAP solution was visually clear with brown particles and filaments, contrasting with the aqueous solution.

### 4.1. Driving Effects of the Migration Electric Field

The concentration of Li^+^ in the organic solution fluctuated differently from that in the catholyte with intensifying voltage, but after 12 V, the trend was significantly increasing in both cases (Figure 5a). Li^+^ had an evident migration rate in the catholyte only after 9 V. The separation effect could be observed differently on each membrane, and the results need to be understood based on their peculiarities. It was noted that AI-1.2 had, from the very beginning, values higher than 1. Figure 5b shows that for AI-1.2, the catholyte–organic solution interface was permanently positive, increasing to 9 V, followed by a constant decrease. The Δ^6^Li value was constantly upbeat, indicating an enrichment in the organic solution. Figure 5c shows that after 9 V, there was a jump, documenting a positive effect of growing voltage after this threshold, reaching values of α = 1.021 (9 V), α = 1.029 (12 V), and α = 1.0317 (15 V). When evaluating the performance of membrane AI-1.3, which also presented only positive Δ^6^Li values, the decrease was slow from 3 V to 15 V. In this case, the effect of voltage was noted at 6 V. AI-1.2 and AI-1.3 were identical; only the organic solution in the anode chamber differed by, respectively, 0.5 mol/L and 1 mol/L LiNT*f*_2_ in the TBAP solution. For AI-2.2, a membrane similar to AI-1.3 (differing in that tetra butyl ammonium perchlorate (TBAP) had a concentration of 0.1M in CH_3_CN), it was noted that the α values were below 1, even if the trend constantly increased. For AI-2.2, the Δ^6^Li values were, in each case, negative, slowly decreasing from 3 V to 15 V. Evaluating these three cases, it can be stated that an organic solution of 1 mol/L shows a low separation factor, and Li^7^ is advantageous for enrichment.

The impregnated membranes before electromigration with double the amount of crown ether (ratio 2:1; AI-2) demonstrated lower enrichment than those with a single crown ether structure (ratio 1:1; AI-1). In all membranes, the crown ether concentration in the ionic liquid within the transfer tube from anode to cathode remained constant at 0.2 mol/L. Similar experiments have shown that crown ether improves ^6^Li ion migration performance [23,27,32]. However, Li^+^ migration from the anode solution to the organic solution remains limited, with negligible enrichment. Research on the separation of Li^+^ from aqueous to organic solutions using ionic liquid electromigration indicated consistent outcomes. It highlighted that the migration efficiency of Li^+^ in the organic phase is low, which is attributed to the increasing concentration gradient between the cathode and anode [37]. The increased concentration difference at the cathode first raises the Li^+^ concentration, followed by a decrease, though higher voltages still impact migration [3]. The experimental results show that the separation coefficient is conditioned by cathode ion mobility and high electrical potential [25]. In addition, a similar study found that 16 V caused a higher enrichment of ^6^Li in the catholyte due to the weakening of the stability of crown ether [37].

### 4.2. Driving Effects of Migration Time

Figure 6a illustrates the concentration of Li^+^ in the organic solution and the changes at the cathode when considering the migration time. In the first 6 h, the growth was slow and became faster at 20 h, after which the trend grew but with reduced intensity. The Li^+^ migration in the organic solution decreased in the first 6 h and increased by 20 h (Figure 6b). The enrichment in Li^+^ concentration in the catholyte decreased at 6 h, after which it increased strongly until 25 h, resulting in another reduction. After the first 6 h, the Δ^6^Li values indicated an enrichment, which was evident until 48 h and was more apparent for AI-1.2 (Figure 6c). The negative values in AI-1.1 document that the use of this system slowly increased Δ^7^Li.

The separation factor was above 1, with slow, sustained growth only in the case of the AI-1.2 membrane (Figure 6d). The experiment that included the AI-1.3 membrane showed that Δ^6^Li had only positive values with a slight decrease, mainly until 20 h, indicating that the enrichment of ^6^Li occurred in the cathode chamber. The separation factor was initially α = 0961 at the 20 h overhead threshold of 1 (α = 1.003), and a maximum of α = 1.021 was achieved at 48 h. A different pattern was observed in the case of the AI-1.1 membrane, which mainly decreased, and at 48 h, the separation factor was below 1 (α = 0.998), documenting that the ^6^Li enrichment was weakening. According to the presented results, there was a change in the enrichment factor at around 20 h in the catholyte. A notable increase in the migration potential of ^6^Li was observed before 6 h, alongside the potential for accumulation in the catholyte, which was influenced by the molar concentration of the organic solution. Previous reports have indicated that Li^+^ derived from the catholyte originates from an organic solution [33]. Furthermore, variations are anticipated due to the combined effects of voltage, diffusion, chelation by the crown ether, and the migration duration, which collectively facilitate an increase in the concentration of Li^+^ in the catholyte [35]. When assessing the combined effect of voltage and migration time, it is worth noting that crown ether can be assigned comparable significance. This is particularly true for the highly impregnated AI-2 membrane, where the Li isotope ions were almost inseparable from the crown ether. In the literature, the opposite process was mentioned, where the crown ether was found to have minimal importance [8]. Even so, regarding our preliminary findings after testing various impregnations, we can state that controlled membrane impregnation helps minimize cross-contamination between isotopes and increases enrichment efficiency.

## 5. Conclusions

In this study, we developed and tested two groups of laboratory-made membranes, non-impregnated membranes before electromigration with Li[NTf2 (AI-1) and impregnated membranes (AI-2). The membranes were characterized under electromigration conditions within a Li salt solution–organic and solution–inorganic system, and the results indicated a better performance for those that were non-impregnated. The optimization process documented that shear viscosity, which was influenced by temperature variations, insignificantly affected Li separation efficiency through electromigration. The main conclusions are as follows: (i) the voltage, migration time, and concentration of tetra butyl ammonium perchlorate (TBAP) with a concentration of 0.1 M in CH_3_CN highly improved the separation factor (α) of the catholyte organic solution system and the enrichment of Li^+^ in the catholyte; (ii) the migration of Li^+^ in the catholyte environment was evident after 9 V, with a migration time that increased from a minimum of 6 h and that differed with different concentrations of organic solution in the anode chamber; (iii) hyper impregnation of membranes with crown ethers (AI-2) increases the migration but not necessarily the separation, mainly in the first hours and when applying high voltage; (iv) an equilibrium between voltage and migration time must be considered and developed dynamically during separation. Since the testing was performed together with several organic and electromigration solutions, it was evident that the separation effect varied significantly. Based on our results, we can state that (i) the membranes exhibited varying performance degrees under different experimental conditions as a result of significant interactions between all factors evaluated, and (ii) by adjusting and combining different parameters, as in this experimental design, the impact of external factors (voltage, migration time, organic solution) on membrane proprieties and separation efficiency must be adjusted in the laboratory to obtain the maximum performance. This approach shows promise for achieving higher levels of ^6^Li enrichment by optimizing impregnation methods and conditions, which is valuable for a range of scientific and industrial applications.

## Figures and Tables

**Table 1:** Experimental conditions under which the optimization tests of the electrochemical lithiation process were carried out (C[M] = molar concentration, E[V] = electric potential). The ionic liquid NT*f*_2_ (1-butyl-3-methylimidazoliu bis((trifluoromethyl)sulfonyl)imide) was mixed with anisole in a 7:3 ratio *v*/*v*, and 0.2 mol/L of 15-crown-5 ether was added to create the organic solution, which was then placed in the intermediate tank between the anode and cathode. Both membranes (AI-1 and AI-2) were exposed to ionic liquid and crown ether in synthesis, respectively, while the AI-2 membranes were exposed to double the amount of crown ether at a ratio of 2:1 (in the first manufacturing stage).

No. Exp.		Membrane	C[M]_Li_	E[V]	Current Range [nA]
1	non-impregnated	AI-1.1 (handmade membrane impregnated in synthesis with 15-crown-5 and dibutyl phthalate (DBP), ratio of 1:1)	1 mol/L Li[NT*f*_2_] in 0.1 M TBAP/CH_3_CN	3–15	(−30)–(+30)
2		AI-1.2 (handmade membrane impregnated in synthesis with 15-crown-5 and dibutyl phthalate (DBP), ratio of 1:1)	0.5 mol/L Li[NT*f*_2_] in 0.1 M TBAP/CH_3_CN	3–15	(0)–(+1)
3		AI-1.3 (handmade membrane impregnated in synthesis with 15-crown-5 and dibutyl phthalate (DBP), ratio of 1:1)	1 mol/L Li[NT*f*_2_] in 0.1 M TBAP/CH_3_CN	3–15	(−3)–(+3)
4	impregnated	AI-2.1 (handmade membrane impregnated in synthesis with 15-crown-5 and dibutyl phthalate (DBP), ratio of 2:1)	0.5 mol/L Li[NT*f*_2_] in 0.1 M TBAP/CH_3_CN	3–15	(0)–(+1)
5		AI-2.2 (handmade membrane impregnated with 15-crown-5 and dibutyl phthalate (DBP), ratio of 2:1)	0.5 mol/L Li[NT*f*_2_] in 0.1 M TBAP/CH_3_CN	3–15	(−45)–(+45)

## Data Availability

The original contributions presented in this study are included in the article. Further inquiries can be directed to the corresponding author.

## References

[1] Yang J., Qu G., Liu C., Zhou S., Li B., Wei Y. (2022). An effective lithium ion-imprinted membrane containing 12-crown ether-4 for selective recovery of lithium. Chem. Eng. Res. Des..

[2] Liang Q., Zhang E.-H., Yan G., Yang Y.-Z., Liu W.-f., Liu X.-G. (2020). A lithium ion-imprinted adsorbent using magnetic carbon nanospheres as a support for the selective recovery of lithium ions. New Carbon Mater..

[3] Ju H., Wang C., Meng Q., Mao L., Zhou X., Zhang P., Xue Z., Shao F., Jing Y., Jia Y. (2024). Electromigration separation of lithium isotopes: The effect of ionic liquid ratios. J. Mol. Liq..

[4] Evans J.A., DeHart M.D., Weaver K.D., Keiser D.D. (2022). Burnable absorbers in nuclear reactors—A review. Nucl. Eng. Des..

[5] Liu B., Jia Y., Zhang Z., Sun H., Yao Y., Jing Y., Qi M., Zhang Q. (2021). Separation of lithium isotopes by crown ether-room temperature ionic liquid-anisole friendly solvent system. J. Mol. Liq..

[6] Sun D., Zhu Y., Meng M., Qiao Y., Yan Y., Li C. (2017). Fabrication of highly selective ion imprinted macroporous membranes with crown ether for targeted separation of lithium ion. Sep. Purif. Technol..

[7] Zhu W., Jia Y., Sun J., Zhang P., Shao F., Jing Y. (2020). Lithium isotope separation using 4′-acetylbenzo-15-crown-5 and 1-butyl-3-methylimidazolium bis[(trifluoromethyl) sulfonyl] imide in the synergistic extraction system. J. Mol. Liq..

[8] Wang M., Sun J., Zhang P., Huang C., Zhang Q., Shao F., Jing Y., Jia Y. (2020). Lithium isotope separation by electromigration. Chem. Phys. Lett..

[9] Murali A., Zhang Z., Sarswat P.K., Free M.L. (2019). Measurements and simulations of Li isotope enrichment by diffusion and electrochemical migration using gel-based electrolyte. Sep. Purif. Technol..

[10] Hoshino T., Terai T. (2011). Basic technology for 6Li enrichment using an ionic-liquid impregnated organic membrane. J. Nucl. Mater..

[11] Sun X.-L., Zhou W., Gu L., Qiu D., Ren D.-H., Gu Z.-G., Li Z. (2015). Liquid–liquid extraction to lithium isotope separation based on room-temperature ionic liquids containing 2,2′-binaphthyldiyl-17-crown-5. J. Nucl. Sci. Technol..

[12] Sun H., Jia Y., Liu B., Jing Y., Zhang Q., Shao F., Yao Y. (2019). Separation of lithium isotopes by using solvent extraction system of crown ether-ionic liquid. Fusion Eng. Des..

[13] Chen S., Dong Y., Sun J., Gu P., Wang J., Zhang S. (2023). Ionic liquids membranes for liquid separation: Status and challenges. Green Chem..

[14] Xiao J., Jia Y., Shi C., Wang X., Yao Y., Jing Y. (2016). Liquid-liquid extraction separation of lithium isotopes by using room-temperature ionic liquids-chloroform mixed solvent system contained benzo-15-crown-5. J. Mol. Liq..

[15] Murali A., Zhang Z., Free M.L., Sarswat P.K. (2021). A Comprehensive Review of Selected Major Categories of Lithium Isotope Separation Techniques. Phys. Status Solidi (a).

[16] Mallah M., Davoudi M. (2012). Evaluation of lithium separation by dispersive liquid–liquid microextraction using benzo-15-crown-5. J. Radioanal. Nucl. Chem..

[17] Hoshino T., Terai T. (2011). High-efficiency technology for lithium isotope separation using an ionic-liquid impregnated organic membrane. FUSION Eng. Des..

[18] Dong Y., Zhu Q., Zou W., Fang J., Yang Z., Xu T. (2023). Dibenzo-15-crown-5-based Tröger’s Base membrane for 6Li+/7Li+ separation. Sep. Purif. Technol..

[19] Zhang Z., Murali A., Sarswat P.K., Free M.L. (2020). High-efficiency lithium isotope separation in an electrochemical system with 1-butyl-3-methylimidazolium dicyanamide, 1-ethyl-3-methylimidazolium bis(trifluoromethylsulfonyl)imide, and diethyl carbonate as the solvents. Sep. Purif. Technol..

[20] Zhou W., Sun X.-L., Gu L., Bao F.-F., Xu X.-X., Pang C.-Y., Gu Z.-G., Li Z. (2014). A green strategy for lithium isotopes separation by using mesoporous silica materials doped with ionic liquids and benzo-15-crown-5. J. Radioanal. Nucl. Chem..

[21] Zhang Z., Jia Y., Liu B., Jing Y., Yao Y. (2021). Characterization of a Novel Crown Ether System for Lithium Isotope Separation. Ind. Eng. Chem. Res..

[22] Gu L., Sun X., Ren D., Qiu D., Gu Z., Li Z. (2015). Extraction separation of lithium isotopes by using XAD-7 resins impregnated with ionic liquid and benzo-15-crown-5. J. Nucl. Radiochem..

[23] Fang Y., Ha R., Sun J., Liu X., Ding X., Shi W. (2024). Research progress on lithium isotopes separation by chemical exchange with crown ethers decorated materials. Green Energy Environ..

[24] Yan F., Liu Y., Wang M., Yang B., Pei H., Li J., Cui Z., He B. (2018). Preparation of polysulfone-graft-monoazabenzo-15-crown-5 ether porous membrane for lithium isotope separation. J. Radioanal. Nucl. Chem..

[25] Xiao J., Jia Y., Shi C., Wang X., Wang S., Yao Y., Jing Y. (2017). Lithium isotopes separation by using benzo-15-crown-5 in eco-friendly extraction system. J. Mol. Liq..

[26] Arienzo I., Liotta M., Brusca L., D’Antonio M., Lupone F., Cucciniello C. (2020). Analytical Method for Lithium Isotopes Determination by Thermal Ionization Mass Spectrometry: A Useful Tool for Hydrogeochemical Applications. Water.

[27] Pei H., Yan F., Liu H., He B., Li J. (2024). The selective complexation of crown ethers for lithium isotope separation: A critical review. Sep. Purif. Technol..

[28] Zhao B., Yin D., Gao Y., Ren J. (2022). Concentration dependence of yield stress, thixotropy, and viscoelasticity rheological behavior of lithium-ion battery slurry. Ceram. Int..

[29] Ouyang L., Wu Z., Wang J., Qi X., Li Q., Wang J., Lu S. (2020). The effect of solid content on the rheological properties and microstructures of a Li-ion battery cathode slurry. RSC Adv..

[30] Bezdomnikov A.A., Demina L.I., Kuz’mina L.G., Kostikova G.V., Zhilov V.I., Tsivadze A.Y. (2023). Study of Lithium-Extraction Systems Based on Benzo-15-Crown-5 Ether and Alkylimidazolium-Based Ionic Liquid. Molecules.

[31] Xiao M., Fan K., Zhang M., Du B., Ran Z., Xing Z. (2022). Effect of Cyclic Olefin Copolymer on Dielectric Performance of Polypropylene Films for Capacitors. IEEE Trans. Dielectr. Electr. Insul..

[32] Christy F.A., Shrivastav P.S. (2011). Conductometric Studies on Cation-Crown Ether Complexes: A Review. Crit. Rev. Anal. Chem..

[33] Wang C., Zhang P., Meng Q., Xue Z., Zhou X., Ju H., Mao L., Shao F., Jing Y., Jia Y. (2023). Electromigration separation of lithium isotopes: The effect of electrolytes. J. Environ. Chem. Eng..

[34] Zhao Z., Zhou X., Meng Q., Zhang P., Shao F., Li X., Li H., Mao L., Zheng T., Jing Y. (2024). Electromigration separation of lithium isotopes with B12C4, B15C5 and B18C6 systems. New J. Chem..

[35] Huang C., Sun J., Wang C., Zhang Q., Wang M., Zhang P., Xue Z., Jing Y., Jia Y., Shao F. (2022). Lithium Isotope Electromigration Separation in an Ionic Liquid–Crown Ether System: Understanding the Role of Driving Forces. Ind. Eng. Chem. Res..

[36] Zhao Z., Mao L., Zheng T., Li X., Ye C., Zhang P., Li H., Sun W., Sun J. (2025). Electromigration Separation of Lithium Isotopes with the Benzo-12-Crown-4-Ether (B12C4) System. Separations.

[37] Wang C.M., Zhang P.R., Ju H.Q., Xue Z.X., Zhou X.L., Mao L.J., Shao F., Zou X.W., Jing Y., Jia Y.Z. (2022). Electromigration separation of lithium isotopes: The multiple roles of crown ethers. Chem. Phys. Lett..

